# Neurosyphilis-Induced Psychosis: A Unique Presentation of Syphilis With a Primary Psychiatric Manifestation

**DOI:** 10.7759/cureus.36080

**Published:** 2023-03-13

**Authors:** Zaid Taki El-Din, Humzah Iqbal, Anil Sharma

**Affiliations:** 1 Department of Psychiatry, St. George's University School of Medicine, West Indies, GRD; 2 Department of Internal Medicine, University of California San Francisco Fresno, Fresno, USA; 3 Department of Psychiatry, St. Francis Medical Center, Lynwood, USA

**Keywords:** psychosis, psychotic disorder, tertiary syphilis, syphilis, neurosyphilis

## Abstract

Syphilis is a predominantly sexually transmitted infection caused by the spirochete Treponema pallidum. The infection presents with four different stages and although rare, can lead to behavioral symptoms if not treated in its earliest form. It can cause psychosis, mania, depression, anxiety, and personality changes. Screening and early treatment of syphilis are essential in preventing neurosyphilis and its neuropsychiatric symptoms. Neurosyphilis is rarely the initial presentation of syphilis. This is a case report of a 30-year-old female with neurosyphilis who presented with psychosis as the primary presentation.

## Introduction

Primary syphilis is a sexually transmitted infection (STI) that presents with a localized painless chancre, typically on the genitals. Secondary syphilis is characterized by a disseminated disease with constitutional symptoms, maculopapular rash, condylomata lata (smooth, painless, wart-like white lesions on genitals), and lymphadenopathy. The first two stages are followed by a latent, asymptomatic phase known as latent syphilis that can either resolve or progress to the tertiary stage leading to multi-organ dysfunction and central nervous system (CNS) abnormalities known as neurosyphilis [1]. Neuropsychiatric manifestations of syphilis are rare, due to the widespread use of penicillin and its efficacy in treating the disease. Patients most often present in an early stage of syphilis, and only 10%-15% of cases progress to tertiary syphilis [2]. Of these cases, less than 20% present with psychiatric symptoms, which can include paranoia, behavioral changes, hallucinations, mania, and cognitive impairment [2,3]. Psychosis as the initial presentation of syphilis is exceedingly rare and has only been reported in a handful of cases. We present a unique case of neurosyphilis diagnosed during an episode of psychosis.

## Case presentation

A 30-year-old female patient was admitted to the hospital with a four-month history of suicidal ideation, anxiety, personality changes, and psychosis. The patient strongly believed that her family members and relatives were monitoring all of her movements and planning to kill her. She strongly suspected they were involved in a big conspiracy against her. She also reportedly engaged in violent behavior and aggression towards family members over the past several months. The patient's family reported that the patient's sleep had been disturbed, sometimes only sleeping three hours a night. The patient denied any drug or alcohol usage and denied any sexual activity for the past several years. Mental status examination (MSE) showed a disheveled young woman with pressured speech who had avoidant eye contact and was restless throughout the interview. The patient had an irritable mood, poor attention, and concentration. She had auditory hallucinations, delusions of persecution, and poor insight and judgment. She reported hearing "the man from the church" tell her "that she is a bad person" but denies suicidal or homicidal ideation. Per family, she was calm and amiable prior to a year ago. She became more easily agitated and angry, with some outbursts leading to physical altercations with family members. These altercations were unprovoked and followed an event "that she imagined in her head" per family. A careful review of the patient’s history revealed no history of psychiatric conditions and no history of STI.

**Figure 1:**
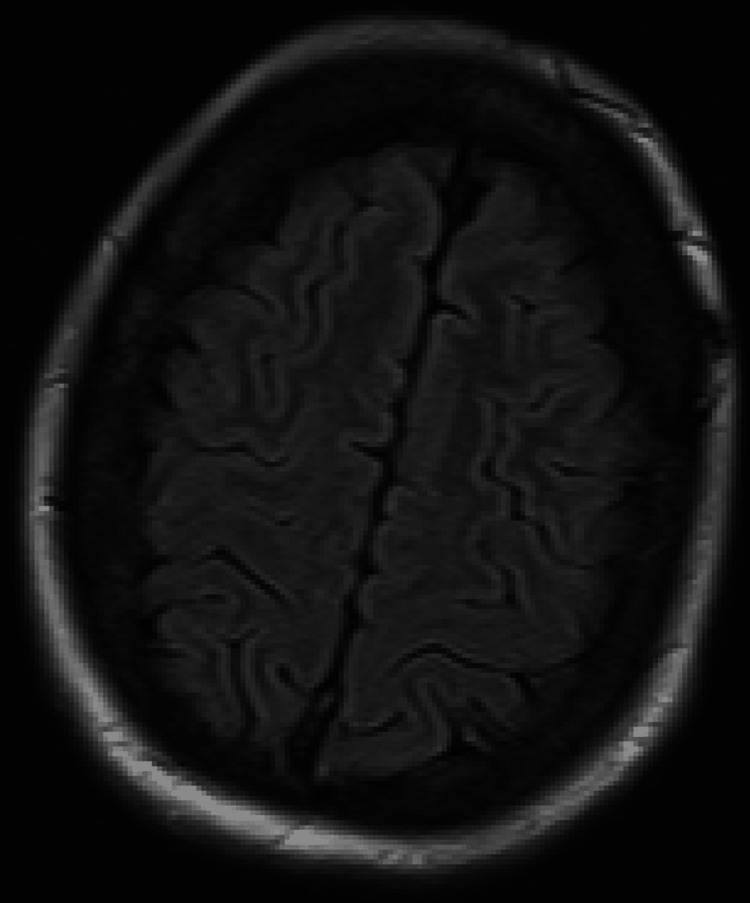
MRI head without contrast demonstrating no evidence of infarction, mass, or lesion as a potential cause of the patient’s symptoms.

**Figure 2:**
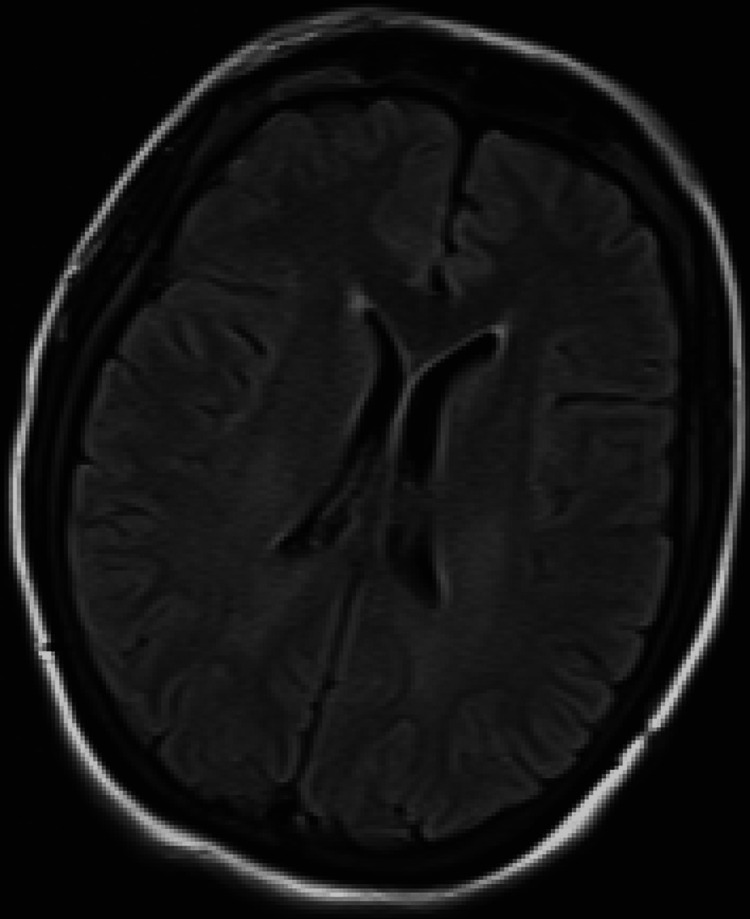
MRI head without contrast demonstrating no evidence of infarction, mass, or lesion as a potential cause of the patient’s symptoms.

The patient’s vital signs were within normal limits and the physical exam was unremarkable aside from MSE findings. Routine investigation of complete blood count, complete metabolic panel, urea, liver function tests, ammonia, urinalysis, thyroid profile, vitamin B12, and folic acid were all within normal limits. The urine drug screen and human immunodeficiency virus (HIV) screen were negative. A magnetic resonance imaging (MRI) scan of the head was unremarkable (Figures 1, 2).

An initial diagnosis of schizophreniform disorder with a possibility of schizophrenia given the time course given by family was unclear was presumed based on the history taken from the patient, family, and MSE. However, Venereal Disease Research Laboratory (VDRL) test returned a positive 1:16 titer, suggesting neurosyphilis as a potential cause. A lumbar puncture was performed, and the cerebrospinal fluid analysis also had a positive VDRL titer, confirming the diagnosis of neurosyphilis. The patient was administered Penicillin G intravenous (IV) for a period of 14 days, during which we observed improvement in the patient's condition. The patient's mood stabilized, agitation decreased, and they reported feeling less anxious and distressed.

## Discussion

Neurosyphilis is observed during tertiary syphilis, which usually presents 5-20 years after initial infection and can have a variety of neurological and psychiatric manifestations [4]. Neurosyphilis itself is rare and most cases of syphilis do not progress to this stage as they are diagnosed early and treated effectively with penicillin. Patients with neurosyphilis may present with tabes dorsalis, Argyll-Robertson pupils, lancinating pains, and uncommonly with psychiatric symptoms including psychosis, mood changes, and dementia [4]. Our patient had a unique presentation of syphilis which presented initially with psychosis, creating a challenging diagnosis. Syphilis with psychosis as the primary presentation is rare and has only been reported sparingly in the literature [5-7]. Our patient met the criteria for the diagnosis of schizophreniform disorder, and without screening for syphilis would likely have entered into a cycle of repeated anti-psychotic treatment, hospital readmission, and worsening of symptoms as seen in cases of syphilis-induced psychosis misdiagnosed as schizophrenia [3]. In cases of schizophrenia-like psychosis with positive testing for syphilis, it may be difficult to determine whether it is an incidental finding or the primary etiology of the presentation. Studies have shown that patients with diagnosed schizophrenia may be at higher risk for acquiring syphilis, which makes a detailed history crucial in the evaluation of these patients [8]. Our patient had no previous psychiatric history prior to this episode, making concurrent schizophreniform/schizophrenia and incidental syphilis infection unlikely, and leaving neurosyphilis as the most likely cause. Clinicians should have a high degree of suspicion for neurosyphilis and take appropriate screening measures when evaluating patients with psychosis in order to initiate timely management.

## Conclusions

In this case report, we share a unique presentation of a 30-year-old female with a four-month history of suicidal ideation, anxiety, personality changes, and psychosis, later diagnosed as neurosyphilis. Syphilis with psychosis as the primary presentation is rare. This case emphasizes the importance of early screening and treatment of syphilis to prevent the progression to neurosyphilis and its neuropsychiatric symptoms. It is essential for healthcare providers to consider medical conditions in their differential diagnosis when evaluating patients with psychosis, as prompt and accurate diagnosis and treatment can significantly improve patient outcomes.
